# Post-Office Matters

**Published:** 1869-02

**Authors:** 


### POST-OFFICE MATTERS.

WHILE the post-office department is endeavoring to make it pay its own expenses, a large proportion of business-men and others are cheating the department, either designedly or inadvertently, in a thousand different ways; and for the benefit of our exchanges and readers a statement will now be made which will astonish some and make many feel, if they do not express the sentiment, "it is an outrage" for Uncle Sam to be so particular. Two different pieces of printed matter cannot be sent in one envelope, even if prepaid, without violating the postal revenue. You may send as many of the same newspaper or pamphlet as will weigh four ounces for two cents, but not two different newspapers or two different pamphlets, or a newspaper and a pamphlet, even if both will not weigh half an ounce. A publisher cannot send an advertisement, even if relating to his own business, in his own paper, if it is on a different piece of paper, nor can an advertisement be stitched in a magazine unless it is a part of the magazine itself, of the same color, and a part of the sheet.

In other words, the laws and regulations of the department will not allow two or more different kinds of mailable matter to be sent in one wrapper or envelope at one rate. Circulars, advertisements, or any other matter not included in the regular issue, must be mailed separately, and paid for at the rates prescribed by law.

According to this ruling a newspaper and a magazine cannot be sent by mail for two cents, although both weigh less than four ounces; nor can two newspapers be sent for one postage; and we know that cart-loads of such matter are thrown aside as waste paper in the New York City Post-office.

This will explain to a vast multitude who send papers, etc., to their friends in the country why they are never received.

These seem to be stringent rules, but it all shows the homely fact, expressed in homely phrase, that while the post-office department is doing all in its power to make it pay its own expenses, the people are trying every possible way to cheat the department out of their legal dues. How many readers can say that they have never written a word on the margin of a newspaper sent to a friend? How many of them can say they have not violated the "rulings" above referred to by sending two pieces of printed matter in one envelope? While we give credit to the gentlemen connected with the post-office for their fidelity and watchfulness, we think the law ought to be so altered that four ounces of printed matter ought to pass through the mails for two cents, whether it be made up of one or fifty pieces of printed paper; and doubtless that was the intention of the framers of the present law.

